# Inhibition of mammalian target of rapamycin by rapamycin increases the radiosensitivity of esophageal carcinoma Eca109 cells

**DOI:** 10.3892/ol.2014.2186

**Published:** 2014-05-27

**Authors:** DEJUN ZHANG, JIE XIANG, YUMING GU, WEI XU, HAO XU, MAOHENG ZU, DONGSHENG PEI, JUNNIAN ZHENG

**Affiliations:** 1 Department of Interventional Radiology, Affiliated Hospital of Xuzhou Medical College, Xuzhou, Jiangsu 221002, P.R. China; 2 Department of Rehabilitation Medicine, Affiliated Hospital of Xuzhou Medical College, Xuzhou, Jiangsu 221002, P.R. China; 3 Jiangsu Key Laboratory of Biological Cancer Therapy, Xuzhou Medical College, Xuzhou, Jiangsu 221002, P.R. China

**Keywords:** esophageal carcinoma, mammalian target of rapamycin, rapamycin, radiosensitization, DNA damage repair

## Abstract

The aim of the present study was to investigate whether radiation induces the mammalian target of rapamycin (Rap) (mTOR) signaling pathway in esophageal carcinoma Eca109 cells, and whether mTOR inhibition by rapamycin increases Eca109 cell radiosensitivity. Changes in the levels of mTOR signaling pathway and DNA damage-repair proteins in Eca109 cells prior to and following radiation were determined. The Eca109 cells were treated with Rap (0, 100, 200 and 400 nmol/l) in combination with radiation (0, 2, 4 and 6 Gy). The cell proliferation inhibition rate was determined by MTT assay. The optimum Rap concentration and radiation dose, which appropriately inhibited cell proliferation, were then selected for further study. An appropriate combination of Rap and radiation for the Eca109 cells was also selected and changes in the mTOR signaling pathway, apoptosis and DNA damage-repair proteins, as well as in cell clone formation, survival curves, the apoptosis rate and radiation-induced DNA damage were determined. The expression of the mTOR signaling pathway and DNA damage-repair proteins were found to increase following the irradiation of the Eca109 cells. In addition, Rap was found to inhibit the mTOR signaling pathway and the expression of the DNA damage-repair proteins. At the same radiation dose, with increasing Rap concentration, the proliferation inhibition rates of the Eca109 cells were found to improve. The clone formation and survival curves of the experimental group were less than those of the control groups. Furthermore, the cell apoptosis rate and expression of cleaved caspase-3 and bax in the experimental group were higher than those of the control groups, whereas the expression of bcl-2 was less than that of the control groups. The radiation-induced DNA damage of the experimental group was greater than that of the control group. The inhibition of mTOR by Rap was found to effectively inhibit the proliferation, survival and radiation-induced DNA damage repair of the Eca109 cells following irradiation, as well as promoting radiation-induced apoptosis, thereby increasing the radiosensitivity of the esophageal carcinoma Eca109 cells.

## Introduction

The phosphatidylinositol 3-kinase (PI3K)/Akt pathway is a cell survival pathway that is important in cell growth and proliferation (1). In addition, this pathway is known to be activated by radiation. Mammalian target of rapamycin (Rap) (mTOR) is a 289-kDa serine/threonine kinase and a downstream target of Akt (2). The normal activation of mTOR may lead to an increase in protein translation, as mTOR phosphorylates and activates the translation regulators, eukaryotic initiation factor 4E-binding protein 1 and ribosomal p70S6 kinase (3,4). In addition, it has been shown that mTOR is important for the oncogenic transformation induced specifically by PI3K and Akt, components of a pathway that has also been indicated to be involved in tumorigenesis (5), which is becoming an important target for cancer treatment (6,7).

The PI3K/Akt pathway has also been demonstrated to be associated with the occurrence, development and prognosis of esophageal carcinoma. Hou *et al* (8) reported that the overexpression of mTOR signaling in esophageal carcinoma Eca109 and EC9706 cells was found to positively correlate with the malignancy of cancer cells. In addition, Hirashima *et al* (9) reported that mTOR signaling was abnormally activated in 116/167 (69.5%) cases of esophageal squamous cell carcinoma (ESCC) in five ESCC cell lines. Clinically, Hirashima *et al* (10) also reported that the overexpression of phosphorylated (p)-mTOR was an independent factor associated with a poor prognosis in esophageal carcinoma. Furthermore, Hildebrandt *et al* (11) reported that gene mutations in the PI3K/Akt/mTOR signaling pathway (Akt1, Akt2 and FRAP1) are associated with the clinical prognosis of chemoradiotherapy.

mTOR has also been investigated as a target for cancer therapy (6,12). Nishikawa *et al* (13) reported that temsirolimus (a rapamycin derivative) treatment reduced the ability of ESCC cells to proliferate, and thus inhibited subcutaneous tumors in nude mice and effectively prolonged the survival of orthotopic esophageal cancer-bearing mice. The mTOR inhibitor was also demonstrated to decrease the phosphorylation of its downstream effectors, and decrease gene expression and protein synthesis, thus, effectively obstructing the pro-growth, pro-proliferation and pro-survival effects of mTOR (14).

It has been reported that the combination of Rap and the DNA-damaging chemotherapeutic agent, cisplatin, may present an effective means of improving cancer treatment (15,16). However, whether mTOR inhibition enhances radiation-induced DNA damage in esophageal carcinoma cells remains unclear. The aim of the present study was to investigate the effects of radiation on mTOR signaling and to determine whether the inhibition of mTOR by Rap enhances the radiosensitivity of Eca109 cells.

## Materials and methods

### Cell culture

The Eca109 cell lines were obtained from Chongqing Medical University (Chongqing, China) and were cultured in Dulbecco’s modified Eagle’s medium (Gibco-BRL, Carlsbad, CA, USA) supplemented with 10% fetal bovine serum (Gibco-BRL), 100 U/ml penicillin and 100 μg/ml streptomycin. All cells were incubated at 37°C in an atmosphere of 5% CO_2_.

### Western blotting

All cells were homogenized in protein lysis buffer (Beyotime Institute of Biotechnology, Nanjing, China) and centrifuged at 15,000 × g for 15 min, and the supernatant was harvested to obtain the total cellular protein extracts. The protein concentrations were determined using the bicinchoninic acid method. The total cellular protein extracts were separated by 6% SDS-PAGE for p-mTOR and DNA-dependent protein kinase catalytic subunit (DNA-PKcs), on 10% SDS-PAGE for p-p70S6K, Ku70, Ku80 and β-actin, and on 12% SDS-PAGE for cleaved caspase-3, bax and bcl-2. The proteins were electrotransferred to nitrocellulose membranes (Amersham Pharmacia Biotech, Stockholm, Sweden) by a wet or semi-dry transfer. The membranes were then blocked with 0.5% skimmed milk and Tris-buffered saline with Tween 20 (TBST) for 2 h at room temperature (RT) and rinsed three times with TBST for 30 min. Next, the cells were incubated with primary polyclonal rabbit anti-human antibodies against p-mTOR, DNA-PKcs, p-p70S6K, p-p70S6K, Ku70, Ku80 and β-actin purchased from Bioworld (Dublin, OH, USA) and cleaved polyclonal rabbit anti-human caspase-3, polyclonal rabbit anti-human bax and polyclonal mouse anti-human bcl-2 purchased from Santa Cruz Biotechnology, Inc., (Santa Cruz, CA, USA) and diluted with 0.5% skimmed milk in TBST at 4°C overnight, followed by rinsing three times with TBST for 30 min. The cells were then incubated with the appropriate monoclonal goat anti-mouse immunofluorescence-conjugated secondary antibodies (Odyssey, Lincoln, NE, USA). Finally, the bands of specific proteins on the nitrocellulose membranes (Amersham Pharmacia Biotech) were visualized with an Odyssey infrared imaging system (Odyssey).

### MTT assay

The cell suspension (200 μl) was seeded in 96-well plates (3,000 cells/well), into five repeat wells and cultured for 24 h. Next, the cells were treated with 0, 100, 200 and 400 nmol/l Rap or the same volume of dimethyl sulfoxide (DMSO) treatment for 1 h, in addition to treatment with different radiation doses of 0, 1, 2, 4 and 6 Gy, followed by a five day incubation period. Next, 20 μl/well of MTT solution (5 g/l; Sigma-Aldrich, St. Louis, MO, USA) was added and the cells were incubated at 37°C for 4 h. The medium was then aspirated and 150 μl DMSO was added and oscillated for 10 min for formazan solubilization. The absorbance was determined at a wavelength of 470 nm using a microplate reader (BioTek Instruments, Inc., Winooski, VT, USA).

### Clonogenic assay

The cell suspensions (2 ml) were seeded in six-well plates (1,000 cells/well), into three repeat wells, and cultured for 24 h, following treatment with 200 nmol/l Rap or the same volume of DMSO for 1 h, and radiation with various doses of 0, 1, 2, 4 or 6 Gy. The cells were then incubated for 10–14 days and fixed with 4% paraformaldehyde (Beijing Solarbio Sciences and Technology Co., Ltd., Beijing, China) and stained with crystal violet (Sigma-Aldrich). The clone formations (≥50 cells) were counted using an Olympus microscope (Olympus, Tokoyo, Japan).

### Fluorescence-activated cell sorting (FACS)

The Eca109 cells treated with a combination of Rap and radiation, and Rap or radiation alone, were trypsinized, washed with cold phosphate-buffered saline (PBS) and resuspended in PBS. A total of 500 μl binding buffer, 5 μl Annexin V-fluorescein isothiocyanate (final concentration of 1 μg/ml) and 5 μl propidium iodide (final concentration of 250 ng/ml) (BD Biosciences, Franklin Lakes, NJ, USA) was added to the mixture. The cells were then vortexed and incubated for 10 min at RT in the dark for flow cytometric analysis using a FACScan Flow Cytometer (BD Biosciences).

### Comet assay

The cell suspension was added to PBS and mixed with low-melting point agarose (200 cells/100 μl) to prepare the slides for the comet assay. The cells were lysed for 2 h in 4°C precooling PBS (pH 8.0–8.4) and the DNA was uncoiled for 20 min in Tris-borate-EDTA buffer. Electrophoresis was conducted at 20 V and 200 mA for 20 min, followed by staining with ethidium bromide (2.5 μg/ml) for 10 sec. The cells were then examined at ×200 magnification using a fluorescence microscope (Nikon Inc., Melville, NY, USA). The tail moment of 50 randomly selected cells per group was measured using comet assay analysis CASP1.2 software (Krzysztof Konca, Wroclaw, Poland).

### Statistical analysis

The experimental data were analyzed using SPSS version 16.0 (SPSS, Inc., Chicago, IL, USA) and quantitative data was presented as χ^2^ ± standard deviation. Two groups were compared using the t-test and multiple groups were compared using one-way analysis of variance. P<0.05 was considered to indicate a statistically significant difference and all P-values were two-sided.

## Results

### Radiation induces mTOR signaling of esophageal carcinoma Eca109 cells, and mTOR inhibitor Rap inhibits this effect

**Figure 1:**
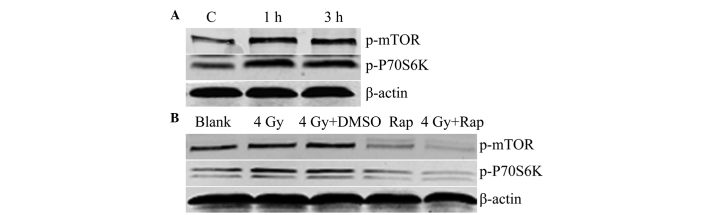
(A) Radiation-induced mTOR signaling (C, control with no radiation; 1 h, 1 h post-irradiation with 4 Gy; and 3 h, 3 h post-irradiation with 4 Gy). (B) Rap inhibited mTOR signaling (blank group, PBS only; 4 Gy group; 4 Gy + DMSO group; Rap group, 200 nmol/l Rap; 4 Gy + Rap group, 4 Gy + 200 nmol/l Rap). mTOR, mammalian target of rapamycin; p-mTOR, phosphorylated mammalian target of rapamycin; DMSO, dimethyl sulfoxide; Rap, rapamycin; PBS, phosphate-buffered saline.

Western blotting revealed that following irradiation with 4 Gy radiation for 1 or 3 h, the expression levels of p-mTOR and p-P70S6K were increased, and were higher than those of the control groups. These results showed that radiation induced mTOR signaling in the Eca109 cells (Fig. 1A). In addition, the combination of Rap and 4 Gy radiation for 1 h was found to decrease the expression of p-mTOR and p-P70S6K in the Eca109 cells of the Rap and 4 Gy + Rap groups, which indicated that the mTOR inhibitor, Rap, inhibits the mTOR signaling pathway, and may also inhibit the radiation-induced mTOR signaling pathway (Fig. 1B).

### mTOR inhibition by Rap improves the proliferation inhibition rate of Eca109 cells treated with radiation

**Figure 2:**
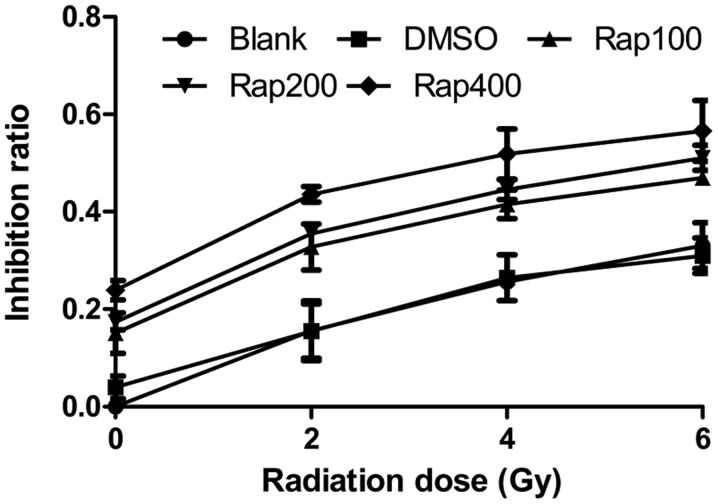
Rap improved the proliferation inhibition rates of Eca109 cells treated with irradiation (blank, PBS only; DMSO, DMSO only; Rap100, 100 nmol/l Rap; Rap200, 200 nmol/l Rap; and Rap400, 400 nmol/l Rap). DMSO, dimethyl sulfoxide; PBS, phosphate-buffered saline; Rap, rapamycin.

The MTT assay showed that following irradiation with 0, 2, 4, and 6 Gy, the proliferation inhibition rates of the Eca109 cells treated with 100, 200 (Rap200) and 400 nmol/l Rap were higher than that of the blank group (P=0.005 for 100 nmol; P=0.001 for 200 nmol; and P<0.001 for 400 nmol) and the DMSO group (P=0.007 for 100 nmol; P=0.001 for 200 nmol; and P<0.001 for 400 nmol) (Fig. 2), while no significant difference was identified between the blank and DMSO groups (P=0.899). Within the same radiation dose with increasing Rap concentrations, the proliferation inhibition rate of the Eca109 cells improved. In addition, within the same Rap concentration with increasing radiation doses, the proliferation inhibition rate also improved. These results showed that the mTOR inhibitor, Rap, inhibits the proliferation of Eca109 cells, and may also inhibit the proliferation of Eca109 cells treated with irradiation.

### mTOR inhibition by Rap decreases the survival fraction of Eca109 cells treated with radiation

**Figure 3:**
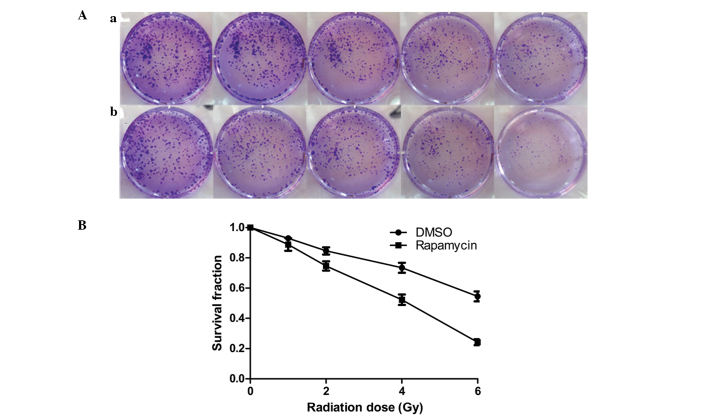
(A) Rap inhibited the clone formation of Eca109 cells treated with (a) radiation + DMSO, and (b) radiation + 200 nmol/l Rap. From left to right the radiation doses were 0, 1, 2, 4 and 6 Gy, respectively. (B) Rap inhibits the survival curves of Eca109 cells treated with radiation. DMSO, dimethyl sulfoxide; Rap, rapamycin.

The clonogenic assay revealed that with increasing radiation doses (0, 1, 2, 4 and 6 Gy) cell clone formation was reduced, and at each radiation dose the clone formation of the Eca109 cells in the radiation + Rap200 groups was less than that of the radiation + DMSO groups (Fig. 3A). In addition, the survival curve for the Eca109 cells in the radiation + Rap200 groups was found to be significantly lower compared with that of the radiation + DMSO groups (P=0.015; Fig. 3B).

### mTOR inhibition by Rap promotes the apoptosis of Eca109 cells treated with radiation

**Figure 4:**
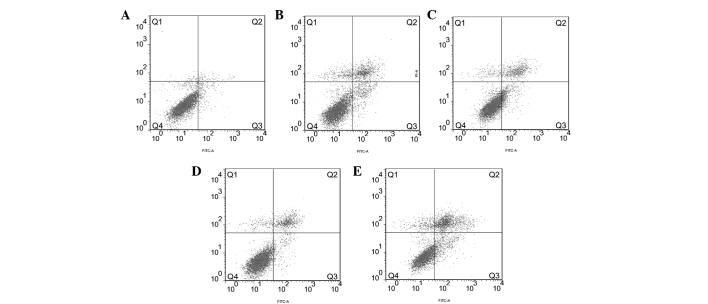
Rap promoted the apoptosis of the Eca109 cells treated with radiation. (A) Blank group, PBS only; (B) 4 Gy group; (C) 4 Gy + DMSO group; (D) 200 nmol/l Rap group; and (E) 4 Gy + 200 nmol/l Rap group. FITC, fluorescein isothiocyanate; Rap, rapamycin; PBS, phosphate-buffered saline; DMSO, dimethyl sulfoxide.

FACS analysis showed that the apoptosis rate of the Eca109 cells in the 4 Gy + Rap200 group (31.63±0.90%) was higher than that of the Rap200 (14.04±0.15%), 4 Gy + DMSO (17.96±0.31%), 4 Gy only (17.35±0.61%) and blank (4.19±0.48%) groups (all P<0.001; Fig. 4). The apoptosis rate of the Rap200 group was lower than that of the 4 Gy + DMSO (P=0.002) and 4 Gy (P=0.001) groups, while no significant difference was identified between the 4 Gy + DMSO and 4 Gy groups.

### mTOR inhibition by Rap promotes the apoptotic protein expression of Eca109 cells treated with radiation

**Figure 5:**
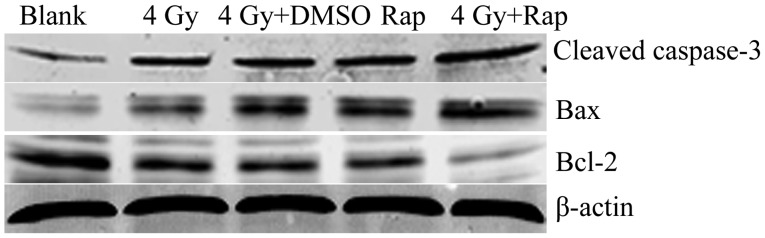
Changes in apoptosis and apoptosis-inhibiting protein expression following mTOR inhibition by Rap (blank group, PBS only; 4 Gy group; 4 Gy + DMSO group; Rap group, 200 nmol/l Rap only; and 4 Gy + Rap group, 4 Gy + 200 nmol/l Rap). DMSO, dimethyl sulfoxide; Rap, rapamycin; PBS, phosphate-buffered saline.

Western blotting revealed that the expression of the apoptotic proteins, cleaved caspase-3 and bax, in the 4 Gy + Rap200 group was significantly higher than that of the Rap only, 4 Gy + DMSO, 4 Gy only and blank groups. In addition, the expression of the apoptosis-inhibiting protein, bcl-2, was less than that of the control groups. These results indicated that Rap may promote radiation-induced apoptotic protein expression and inhibit the apoptosis-inhibiting protein expression in Eca109 cells (Fig. 5).

### Radiation induces the expression of DNA damage repair proteins of esophageal carcinoma Eca109 cells, and mTOR inhibitor Rap inhibits this effect

**Figure 6:**
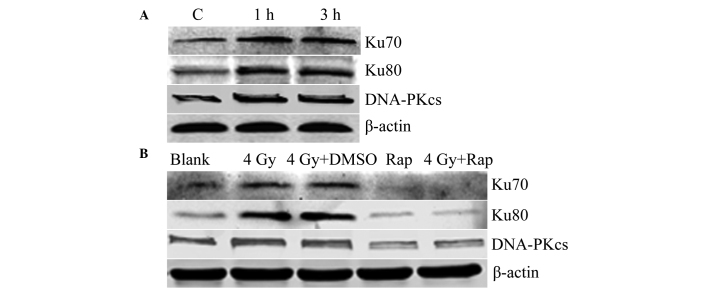
Expression of the DNA damage-repair protein (A) increased following radiation (C, control with no radiation; 1 h, 1 h post-irradiation with 4 Gy; and 3 h, 3 h post-irradiation with 4 Gy) and (B) decreased following mTOR inhibition by Rap (blank group, PBS only; 4 Gy group; 4 Gy + DMSO group; Rap group, 200 nmol/l Rap only; and 4 Gy + Rap group, 4 Gy + 200 nmol/l Rap). DNA-PKcs, DNA-dependent protein kinase catalytic subunit; DMSO, dimethyl sulfoxide; Rap, rapamycin; PBS, phosphate-buffered saline.

The opportunity for DNA damage-repair in cells comes between 4 and 6 h post-radiation treatment. Thus, 1 and 3 h following 4 Gy radiation were selected for analysis. Western blotting showed that the expression of Ku70, Ku80 and DNA-PKcs was increased and higher than that of the control group. These results indicated that radiation induces the expression of the DNA damage-repair proteins in Eca109 cells (Fig. 6A). The combination of Rap and the irradiation of Eca109 cells at 4 Gy for 1 h was found to result in a decrease in the expression of Ku70, Ku80 and DNA-PKcs in the Rap and 4 Gy + Rap groups, which indicated that the mTOR inhibitor Rap inhibits expression of DNA damage-repair proteins, and may also inhibit radiation-induced DNA damage-repair protein expression (Fig. 6B).

### mTOR inhibition by Rap enhances radiation-induced DNA damage in Eca109 cells

**Figure 7:**
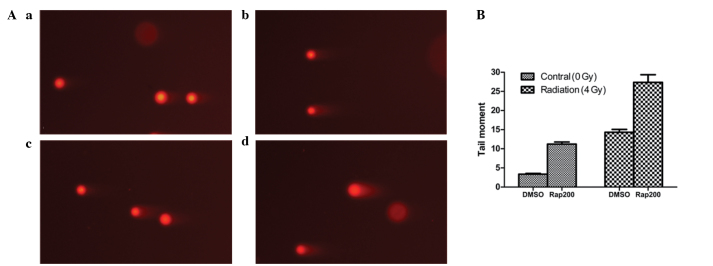
(A) Rap enhanced the radiation-induced DNA damage of the Eca109 cells: (a) DMSO only group; (b) 4 Gy + DMSO group; (c) 200 nmol/l Rap only; and (d) 4 Gy + 200 nmol/l Rap group. (B) Differences between the tail moment in the experimental and control groups. Rap, rapamycin; DMSO, dimethyl sulfoxide; Rap200, 200 nmol/l Rap.

The comet assay revealed that following irradiation with 4 Gy radiation for 30 min, the DNA damage in the Eca109 cells in the 4 Gy + Rap group was greater than that in the control groups (4 Gy + DMSO, Rap200 and DMSO groups; Fig. 7A). Furthermore, the tail moment of the 4 Gy+Rap group (27.35±2.02) was longer than that of the control groups (all P<0.001), whereas the tail moment of the DMSO + 4 Gy (14.32±0.74) and Rap200 (11.21±0.55) groups was longer than that of the DMSO group (3.37±0.20) (both P<0.001; Fig. 7B). These results indicated that the mTOR inhibitor, Rap, may enhance the radiation-induced DNA damage of Eca109 cells.

## Discussion

mTOR, a downstream kinase of the PI3K/Akt pathway, is crucial for cell growth and survival (1). Therefore, the use of mTOR as a therapeutic target is becoming increasingly significant in cancer research. In the present study, radiation was found to increase mTOR signaling in the Eca109 cell line, and the mTOR inhibitor was found to block this signaling activation, enhancing the radiosensitivity of the Eca109 cells.

As radiotherapy is an important adjuvant therapy for esophageal carcinoma, it is undesirable that radiation may contribute to cancer cell survival. In the present study, mTOR signaling was increased by ionizing radiation in the esophageal carcinoma Eca109 cells. Therefore, blocking the activation of radiation-induced mTOR signaling presents a method of enhancing the cytotoxic effects of radiation. To demonstrate this, the Eca109 cells were treated with the mTOR inhibitor, Rap, to block the increase in phosphorylation of the downstream marker p70S6K protein. The results showed that the proliferation inhibition rate of the Eca109 cells treated with a combination of Rap and radiation was increased (Fig. 2), while the survival fraction was decreased (Fig. 3). This indicated that the inhibition of mTOR by Rap may effectively block the pro-survival response of esophageal carcinoma cells to radiation, and that mTOR inhibition may present a method for enhancing the efficacy of radiotherapy.

Hou *et al* (15) used a combination of Rap and the DNA-damaging agent, cisplatin, to treat the subcutaneous tumors of ectopic esophageal carcinoma in nude mice, and revealed that it resulted in greater inhibition of tumor growth, while Rap sensitized the cancer cells to DNA damage-induced apoptosis, which was similar to the effect reported by Beuvink *et al* (16). We hypothesized that combining radiation with the mTOR inhibitor Rap would also effectively sensitize cancer cells to apoptosis. Therefore, the apoptosis rate of the Eca109 cells treated with Rap and radiation was determined. The FACS results revealed that the apoptosis rate of the 4 Gy + Rap200 group was 31.63±0.90, which was found to be significantly higher than that of the Rap200 (14.04±0.15), 4 Gy + DMSO (17.96±0.31), 4 Gy only (17.35±0.61) and blank (4.19±0.48) groups (Fig. 4). In addition, the western blotting results showed that the expression of cleaved caspase-3 and bax were significantly higher than that of the control groups, whereas the expression of bcl-2 was less than that of the control groups (Fig. 5). These results showed that the apoptosis of the cells treated with Rap and radiation was greatly increased, indicating that caspase-dependent and -independent apoptosis contribute to the observed increase in cell apoptosis.

Radiation predominantly results in the death of cancer cells by reducing the number of DNA double-strand breaks (DSBs), however, there are various degrees of DSB repair potency in cells, which affects the radiosensitivity of cancer cells. Two repair pathways have been identified (17): DNA non-homologous end-joining (NHEJ) involving DNA-protein kinases, including Ku70, Ku80 and DNA-PKcs; and homologous recombination involving the ataxia telangiectasia mutated protein. The NHEJ repair pathway is the predominant radiation-induced DNA damage repair pathway in humans (18). In the present study, radiation was found to induce mTOR signaling (Fig. 1A) and simultaneously increase the expression of Ku70, Ku80 and DNA-PKcs following irradiation (Fig. 6A). Therefore, we hypothesized a correlation among the NHEJ repair pathway, radiation and mTOR signaling. The mTOR inhibitor, Rap, was used to inhibit mTOR signaling, and western blotting revealed that when mTOR signaling was obstructed (Fig. 1B), the Ku70, Ku80 and DNA-PKcs expression in the Rap200 and 4 Gy + Rap200 groups was decreased (Fig. 6B). In addition, the comet assay showed that the radiation-induced DNA damage of the 4 Gy + Rap200 group was greater than that of the control groups (Fig. 7A). Furthermore, the tail moment of the 4 Gy + Rap200 group (27.35±2.02) was longer than that of the 4 Gy only (14.32±0.74), Rap200 only (11.21±0.55) and blank (3.37±0.20) groups (Fig. 7B). These results showed that the inhibition of mTOR by Rap also inhibits the NHEJ repair pathway prior to and following radiation, indicating that mTOR inhibition may be a mechanism for decreasing the potency of radiation-induced DNA damage repair, which may contribute to the increased radiosensitivity observed in the present study.

It is known that cell cycle arrest may affect radiosensitivity (19), as cells exhibit varying radiosensitivities in different cell cycle phases, and thus, cell cycle regulation is important for radiosensitivity. The cells are most sensitive to radiation during the G_2_-M phase, less sensitive during the G_1_ phase and least sensitive during the end of the S phase (20). Hou *et al* (15) reported that Rap inhibiting mTOR may result in the cell cycle G_0_/G_1_ arrest of esophageal carcinoma cells. However, this was not investigated in the present study. Whether cell cycle G_0_/G_1_ arrest affects the radiosensitivity of Eca109 cells requires further study.

Additionally, it has been shown that Rap may inhibit angiogenesis (21), and that the mTOR inhibitors, Rap and RAD001, may significantly enhance the radiosensitivity of the tumor vasculature *in vitro* and *in vivo* (22). We hypothesize that mTOR inhibition with Rap may exhibit a greater increase in the radiosensitivity of esophageal carcinoma *in vivo*. It is possible that the combination of mTOR inhibition and radiation may improve the efficacy of tumor radiotherapy via a dual mechanism that promotes radiation-induced tumor cell cytotoxicity and inhibits tumor angiogenesis. Therefore, whether mTOR inhibition enhances the radiosensitivity of esophageal carcinoma *in vivo* also requires further study.

## References

[1] Cantley LC (2002). The phosphoinositide 3-kinase pathway. Science.

[2] Navé BT, Ouwens M, Withers DJ, Alessi DR, Shepherd PR (1999). Mammalian target of rapamycin is a direct target for protein kinase B: identification of a convergence point for opposing effects of insulin and amino-acid deficiency on protein translation. Biochem J.

[3] Beugnet A, Wang X, Proud CG (2003). Target of rapamycin (TOR)-signaling and RAIP motifs play distinct roles in the mammalian TOR-dependent phosphorylation of initiation factor 4E-binding protein 1. J Biol Chem.

[4] Burnett PE, Barrow RK, Cohen NA, Snyder SH, Sabatini DM (1998). RAFT1 phosphorylation of the translational regulators p70 S6 kinase and 4E-BP1. Proc Natl Acad Sci USA.

[5] Nicholson KM, Anderson NG (2002). The protein kinase B/Akt signalling pathway in human malignancy. Cell Signal.

[6] Wong KK, Engelman JA, Cantley LC (2010). Targeting the PI3K signaling pathway in cancer. Curr Opin Genet Dev.

[7] Morgensztern D, McLeod HL (2005). PI3K/Akt/mTOR pathway as a target for cancer therapy. Anticancer Drugs.

[8] Hou G, Xue L, Lu Z, Fan T, Tian F, Xue Y (2007). An activated mTOR/p70S6K signaling pathway in esophageal squamous cell carcinoma cell lines and inhibition of the pathway by rapamycin and siRNA against mTOR. Cancer Lett.

[9] Hirashima K, Baba Y, Watanabe M (2012). Aberrant activation of the mTOR pathway and anti-tumour effect of everolimus on oesophageal squamous cell carcinoma. Br J Cancer.

[10] Hirashima K, Baba Y, Watanabe M (2010). Phosphorylated mTOR expression is associated with poor prognosis for patients with esophageal squamous cell carcinoma. Ann Surg Oncol.

[11] Hildebrandt MA, Yang H, Hung MC (2009). Genetic variations in the PI3K/PTEN/AKT/mTOR pathway are associated with clinical outcomes in esophageal cancer patients treated with chemoradiotherapy. J Clin Oncol.

[12] Gomez-Pinillos A, Ferrari AC (2012). mTOR signaling pathway and mTOR inhibitors in cancer therapy. Hematol Oncol Clin North Am.

[13] Nishikawa T, Takaoka M, Ohara T (2013). Antiproliferative effect of a novel mTOR inhibitor temsirolimus contributes to the prolonged survival of orthotopic esophageal cancer-bearing mice. Cancer Biol Ther.

[14] Emerling BM, Akcakanat A (2011). Targeting PI3K/mTOR signaling in cancer. Cancer Res.

[15] Hou G, Zhang Q, Wang L, Liu M, Wang J, Xue L (2010). mTOR inhibitor rapamycin alone or combined with cisplatin inhibits growth of esophageal squamous cell carcinoma in nude mice. Cancer Lett.

[16] Beuvink I, Boulay A, Fumagalli S (2005). The mTOR inhibitor RAD001 sensitizes tumor cells to DNA-damaged induced apoptosis through inhibition of p21 translation. Cell.

[17] van Gent DC, Hoeijmakers JH, Kanaar R (2001). Chromosomal stability and the DNA double-stranded break connection. Nat Rev Genet.

[18] Lieber MR, Ma Y, Pannicke U, Schwarz K (2003). Mechanism and regulation of human non-homologous DNA end-joining. Nat Rev Mol Cell Biol.

[19] Hwang HS, Davis TW, Houghton JA, Kinsella TJ (2000). Radiosensitivity of thymidylate synthase-deficient human tumor cells is affected by progression through the G1 restriction point into S-phase: implications for fluoropyrimidine radiosensitization. Cancer Res.

[20] Pawlik TM, Keyomarsi K (2004). Role of cell cycle in mediating sensitivity to radiotherapy. Int J Radiat Oncol Biol Phys.

[21] Guba M, von Breitenbuch P, Steinbauer M (2002). Rapamycin inhibits primary and metastatic tumor growth by antiangiogenesis: involvement of vascular endothelial growth factor. Nat Med.

[22] Shinohara ET, Cao C, Niermann K, Mu Y, Zeng F, Hallahan DE, Lu B (2005). Enhanced radiation damage of tumor vasculature by mTOR inhibitors. Oncogene.

